# Genetics of heart rate in heart failure patients (GenHRate)

**DOI:** 10.1186/s40246-019-0206-6

**Published:** 2019-05-21

**Authors:** Kaleigh L. Evans, Heidi S. Wirtz, Jia Li, Ruicong She, Juan Maya, Hongsheng Gui, Andrew Hamer, Christophe Depre, David E. Lanfear

**Affiliations:** 1 Department of Internal Medicine, Henry Ford Hospital, 2799 West Grand Blvd. K-14, Detroit, MI 48202 USA; 2 Amgen Inc, Thousand Oaks, CA USA; 3 Department of Public Health Sciences, Henry Ford Health System, Detroit, MI USA; 4 Center for Individualized and Genomic Medicine Research, Henry Ford Hospital, Detroit, MI USA; 5 Heart and Vascular Institute, Henry Ford Hospital, Detroit, MI USA

**Keywords:** Genetics, Heart rate, African Americans, Single nucleotide polymorphisms

## Abstract

**Background:**

Elevated resting heart rate (HR) is a risk factor and therapeutic target in patients with heart failure (HF) and reduced ejection fraction (HFrEF). Previous studies indicate a genetic contribution to HR in population samples but there is little data in patients with HFrEF.

**Methods:**

Patients who met Framingham criteria for HF and had an ejection fraction < 50% were prospectively enrolled in a genetic HF registry (2007–2015, *n* = 1060). All participants donated blood for DNA and underwent genome-wide genotyping with additional variants called via imputation. We performed testing of previously identified variant “hits” (43 loci) as well as a genome-wide association (GWAS) of HR, adjusted for race, using Efficient Mixed-Model Association Expedited (EMMAX).

**Results:**

The cohort was 35% female, 51% African American, and averaged 68 years of age. There was a 2 beats per minute (bpm) difference in HR by race, AA being slightly higher. Among 43 candidate variants, 4 single nucleotide polymorphisms (SNPs) in one gene (*GJA1*) were significantly associated with HR. In genome-wide testing, one statistically significant association peak was identified on chromosome 22q13, with strongest SNP rs535263906 (*p* = 3.3 × 10^−8^). The peak is located within the gene Cadherin EGF LAG Seven-Pass G-Type Receptor 1 (*CELSR1*), encoding a cadherin super-family cell surface protein identified in GWAS of other phenotypes (e.g., stroke). The highest associated SNP was specific to the African American population.

**Conclusions:**

These data confirm *GJA1* association with HR in the setting of HFrEF and identify novel candidate genes for HR in HFrEF patients, particularly *CELSR1*. These associations should be tested in additional cohorts.

**Electronic supplementary material:**

The online version of this article (10.1186/s40246-019-0206-6) contains supplementary material, which is available to authorized users.

## Introduction

Heart failure (HF) remains a considerable public health problem, with an estimated 5.7 million people living with HF in the USA, resulting in > 1 million HF hospitalizations and > $30 billion in health care costs annually [1]. Chronic HF is associated with repeated hospitalizations, substantially reduced quality of life, and a yearly mortality rate in the USA of 7.5% [2]. Observational and interventional studies in adults demonstrate that elevated heart rate (HR) is a modifiable risk factor in patients with HF [3–6]. Beta-blockers represent one of the most important therapeutic options for reducing morbidity and mortality in HF, which may partly be contributed to by their capacity to reduce HR. More recently, the Systolic Heart treatment with the I_*f*_ inhibitor ivabradine Trial (SHIFT) demonstrated that specifically targeting HR reduction in HF patients with reduced ejection fraction (HFrEF) lowers the risk of hospitalization for worsening HF, establishing HR as not only a marker of risk but a treatment target [6]. Therefore, understanding the genetic and biologic drivers of HR particularly in the setting of HFrEF may aid in understanding HF pathophysiology, improve prognostication, or inform treatment decision-making.

Previous studies have examined genetic factors influencing HR in various settings. It has recently become clear that HR is a heritable trait [7, 8], suggesting important genetic influences. In the context of beta-blocker (BB) therapy, *GRK5* was suspected of influencing this but in one study showed no association of *GRK5* genotype with heart rate [9]. Several GWAS of HR in non-HF populations have been performed [10–14], which have reported a number of significant variants; however, the impact of these loci in HF patients remains uncharacterized; and to our knowledge, the genetic contribution to HR in patients with established HFrEF has not been previously investigated. The overall goal of this study was to test the validity of previous candidate gene associations and to identify novel genetic determinants of HR in a diverse cohort of HFrEF patients via GWAS.

## Methods

### Study population

The study population (*n* = 1060) was from a genetic registry of HF patients collected at the Henry Ford Health System (September 13, 2007 to April 1, 2015). Entry criteria included patients ≥ 18 years of age meeting the Framingham criteria for HF, with a previously measured left ventricular ejection fraction (LVEF) < 50%, who were enrolled in our covered entity (Health Alliance Plan) for at least 1 year prior to the date of registry enrollment. Left ventricular EF < 50% was used as eligibility criteria because at the time of registry initiation, this was considered standard for systolic HF. Since EF cutoffs for HFrEF have subsequently changed and ivabradine is indicated in EF < 35%, we include additional restricted analyses in this group. The study was approved by local ethics committees and participants provided written informed consent.

### Genotyping and imputation

We performed genotyping on all registry participants using the Axiom Biobank array (Affymetrix®). This array contains 600K single nucleotide polymorphisms (SNPs) including: (1) 300K GWAS markers with minor allele frequencies of > 1%, (2) > 250K markers low frequency (< 1%) coding variants from the exome sequencing project, and (3) an additional 50K variants to improve African ancestry coverage (YRI Booster). This array provides excellent coverage of genomic variation, capturing 90% in European ancestry and ~ 80% in African ancestry patients. It also allows for ancestral quantification and admixture mapping. The genotyping and standard quality checks were carried out in a standard accepted fashion. In brief, SNPs with a minor allele frequency less than 0.01 or not in Hardy–Weinberg equilibrium (HWE, *P* < 1 × 10^−7^) in each major ethnicity group were removed. Multi-allelic sites and ambiguous SNPs were also deleted. Additionally, study participant samples with questioned validity due to either sex inconsistency (between reported and genetically inferred) or duplicate genotyping were removed.

The imputation was conducted on the Michigan Imputation Server. The computation engine is Minimac3 (https://genome.sph.umich.edu/wiki/Minimac3), and the reference panel used was 1000 Genome Phase 3 v5 [15]. Following imputation, we assessed accuracy at each SNP as the squared Pearson correlation (*R*^2^) between the masked genotypes and the imputed allele dosages (also known as posterior mean genotypes). We retained variants passing commonly used imputation quality thresholds (*R*^2^ > 0.3, MAF > 0.01, HWE *P* value > 1 × 10^−7^) for use in the subsequent analyses.

### Clinical data

Patient characteristics including demographic, medical, and lifestyle data were collected at registry enrollment via a standardized questionnaire and physical exam and supplemented by using administrative data maintained by the system. Primary data collection via the questionnaire and study staff assessment included age, sex, HR, blood pressure, New York Heart Association class, self-identified race, and co-morbidities. We utilized electronic administrative databases maintained by HFHS (including data from Health Alliance Plan our covered entity), such as patient encounters, medical claims, laboratory data, and pharmacy claims membership files to supplement primary data collection from the patient. This included medical diagnoses established via ICD-9 diagnosis related groupings (DRG). ICD-9 classification does not contain discriminators for permanent vs. paroxysmal atrial arrhythmias. In patients with a history of atrial fibrillation diagnostic/billing codes, diagnosis additional data was collected and reviewed (clinical ECG’s), and additional sensitivity analyses were performed as described below. HR was a single measurement obtained as a resting pulse rate measured by hand or with an automatic blood pressure cuff by a qualified study personnel during physical exam at registry enrollment.

The assessment of possible non-sinus rhythm (see adjusted analyses below) was done by querying all available ECG data in the system for patients with previous diagnoses of atrial arrhythmia, reviewing ECG tracings temporally closest to, and surrounding enrollment date (i.e., both before and after the enrollment date). We classified patients as likely or unlikely to be in non-sinus rhythm, and this was used as an excluding factor in a secondary analysis. To quantify beta-blocker exposure for adjusted analyses, we chose to use the average beta-blocker exposure over 6 months prior to enrollment. This was accomplished using pharmacy claims data to generate a beta-blocker exposure metric as previously described [16]. This metric summarizes exposure (both dose and adherence) of all BB medications as a proportion of target exposure for HFrEF (per consensus guidelines). Regarding individual agent use, among patients on BB carvedilol (39%) and metoprolol succinate (38%) were most frequently used, but there were smaller groups of patients using metoprolol tartrate (18%) or another beta-blocker (4%).

### Statistical analysis

We first sought to assess previously published genetic loci for HR found by other GWAS studies (in non-heart failure patients) and test for significance in our HFrEF cohort. We reviewed literature for GWAS studies published that identified significant loci for HR. We found five publications [10–14] that identified a total of 43 loci (listed in Additional file 1: Table S1). We tested the association of these genotypes using linear regression adjusted for self-identified race and kinship. For this analysis, we used the Bonferroni correction for 43 multiple comparisons, which yields a critical *P* value threshold of 1.16 × 10^−3^.

The primary objective of our study was to identify individual SNPs and genes associated with resting HR, adjusted for race (since this was associated with HR). We used Efficient Mixed-Model Association eXpedited (EMMAX) analyses for genome-wide association analysis [17]. This approach uses a kinship matrix to take into account population structure and relatedness. Associations with *P* < 5 × 10^−8^ were considered genome-wide significant [18]. We performed several adjusted and sensitivity analyses to mitigate the contribution of potential confounders and assess the robustness of our findings. First, we repeated our primary analysis excluding patients that may have had non-sinus rhythm (e.g., atrial fibrillation or flutter) upon enrollment. Next, we performed similar analyses adjusted for beta-blocker exposure (quantified as described above).

We secondarily also took a gene-based analytic approach, combining the data from all SNPs within each gene region using the SNP-set kernel-machine association test (SKAT) [19]. This method uses a logistic kernel-machine model, aggregating individual score test statistics of SNPs, and provides a global *P* value for the set of variants tested that takes into account the joint effect of the SNPs in a given SNP set. Gene regions were defined based upon the GENCODE annotation [20]. Multiple comparisons were accounted for using the false discovery rate method of Benjamini-Hochberg [21].

## Results

The study cohort baseline characteristics are shown in Table 1. Overall, the cohort was 35% female and 51% AA and had an average age of 68 years. We tested HR differences by race which revealed a clinically small but statistically significant difference in HR by race, with African Americans having on average around 2 bpm higher rates (70 ± 13 vs. 72 ± 13 bpm; *P* = 0.0021). As expected, several characteristics varied across the two racial groups, including HF etiology, co-morbidities, and demographics.

**Table 1:** Baseline characteristics of the study cohort

Characteristic	Overall (*N* = 1060)	African American (*N* = 543)	White (*N* = 517)	*P*
Female	374 (35%)	220 (41%)	154 (30%)	0.001
Age	68 (± 12)	65 (± 12)	71 (± 11)	0.001
Ejection fraction (%)	34.9 (± 11)	33.6 (± 11.4)	36.2 (± 10.4)	0.001
Ischemic etiology	608 (57%)	267 (49%)	341 (66%)	0.001
Hx COPD	234 (22%)	114 (21%)	120 (23%)	0.375
Hx CKD	239 (± 23%)	149 (± 27.4)	90 (± 17.4)	0.001
Hx A-Fib	295 (28%)	104 (19%)	191 (37%)	0.001
Hx stroke/TIA	129 (12%)	69 (12.7%)	60 (11.6%)	0.375
Hx diabetes	441 (42%)	250 (46%)	191 (37%)	0.003
Sys BP (mmHg)	129 ± 23	131 ± 24	126 ± 22	0.002
HR (beats per min.)	71.1 ± 13	72.2 ± 13	69.9 ± 13	0.007
NTproBNP (ng/L)	348 (± 374)	336 (± 386)	362 (± 360)	0.001
Creatinine (mg/dL)	1.29	1.36 (± 1.08)	1.17 (± 0.57)	0.003
BB exposure^a^	0.26 (± 0.29)	0.26 (± 0.3)	0.26 (± 0.29)	0.832

^a^This is the proportion of target BB exposure over 6 months in patients taking BB

Identified from previous reports of GWAS of HR, we tested 43 previously implicated loci in our cohort of HFrEF patients. In total, the association with HR for four of these SNPs were replicated in our study, with *P* values ranging from 2.55 × 10^−4^ to 7.75 × 10^−5^ (Bonferroni corrected critical *P* value threshold 1.16 × 10^−3^). All four of these SNPS were within a single region of chromosome 6, within the gene *GJA1* which encodes connexin 43. The SNP ID, *P* value, and original publication citation for these significant loci are listed in Table 2.

**Table 2:** Replication of other GWAS studies in HFrEF patients

Gene	*P* value	SNP	Source
*GJA1*	7.75 × 10^−5^	rs9398652	Eijgelsheim et al. [10], Deo et al. [11]
*GJA1*	9.10 × 10^−5^	rs12110693	Deo et al. [11]
*GJA1*	9.40 × 10^−5^	rs1015451	Kilpelainen [12]
*GJA1*	2.55 × 10^−4^	rs9320841	Deo et al. [11]

### Genome-wide analyses of genotype influence on heart rate

**Fig. 1:**
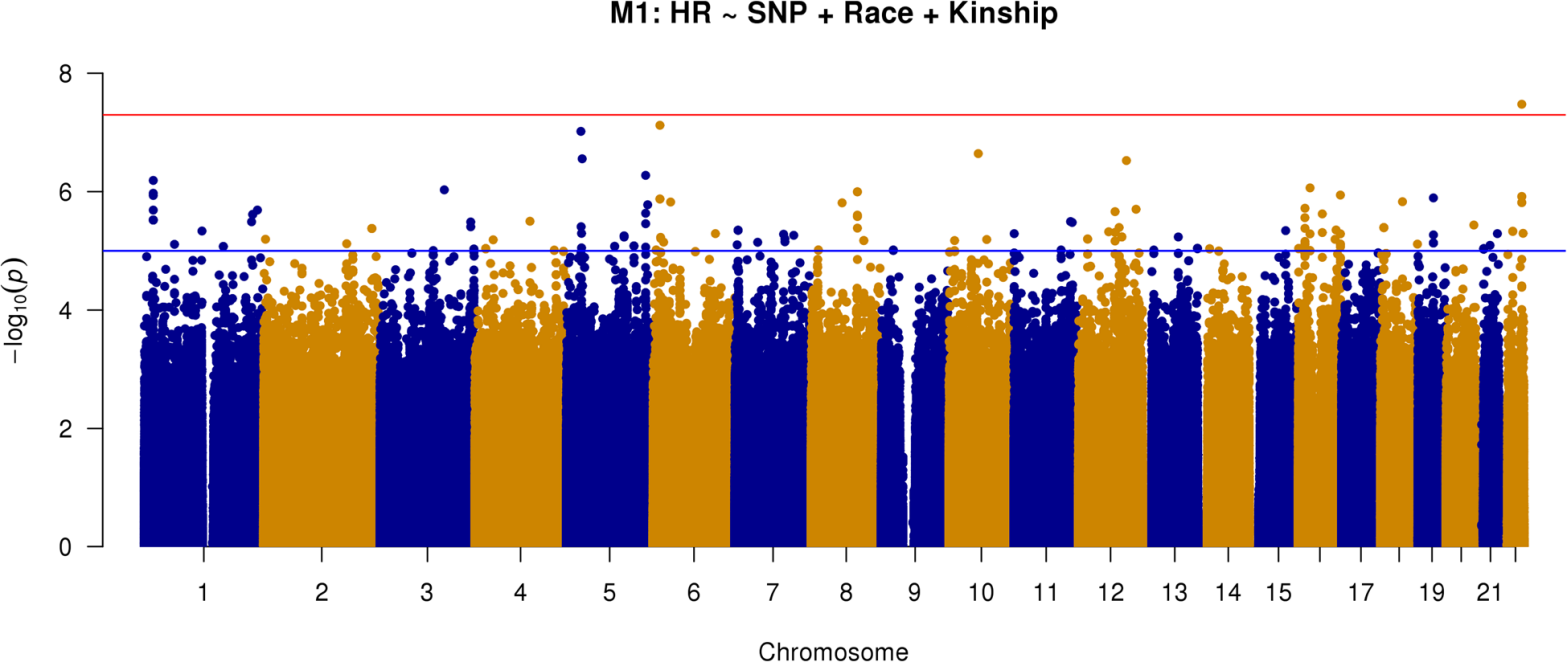
Manhattan plot of GWAS results from model 1: SNP + race + kinship (*n* = 1043; AA = 520, white = 523). Blue reference line: 1 × 10^−5^; red reference line: 5 × 10^−8^

**Fig. 2:**
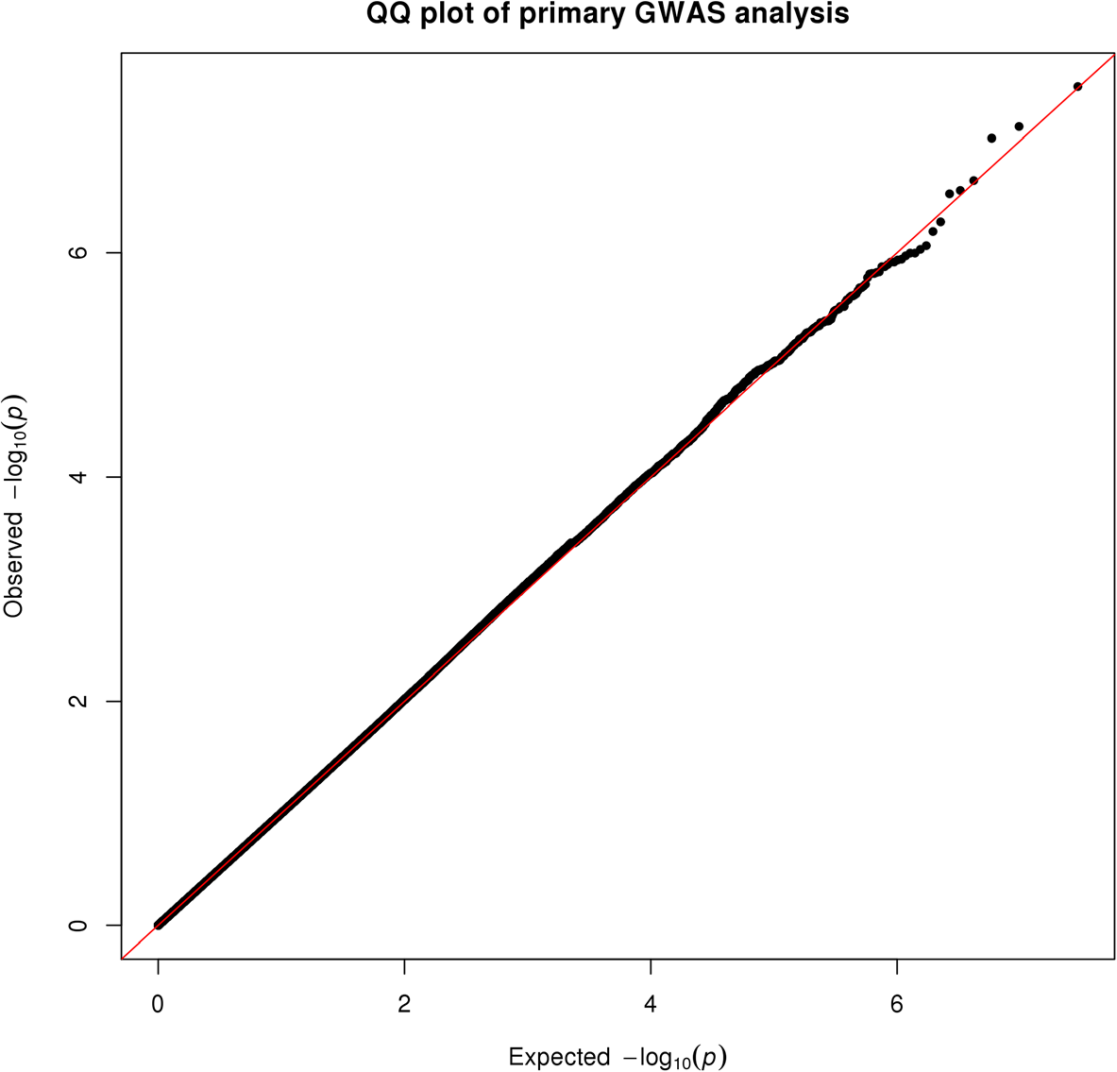
Q-Q plot of primary GWAS analysis

**Fig. 3:**
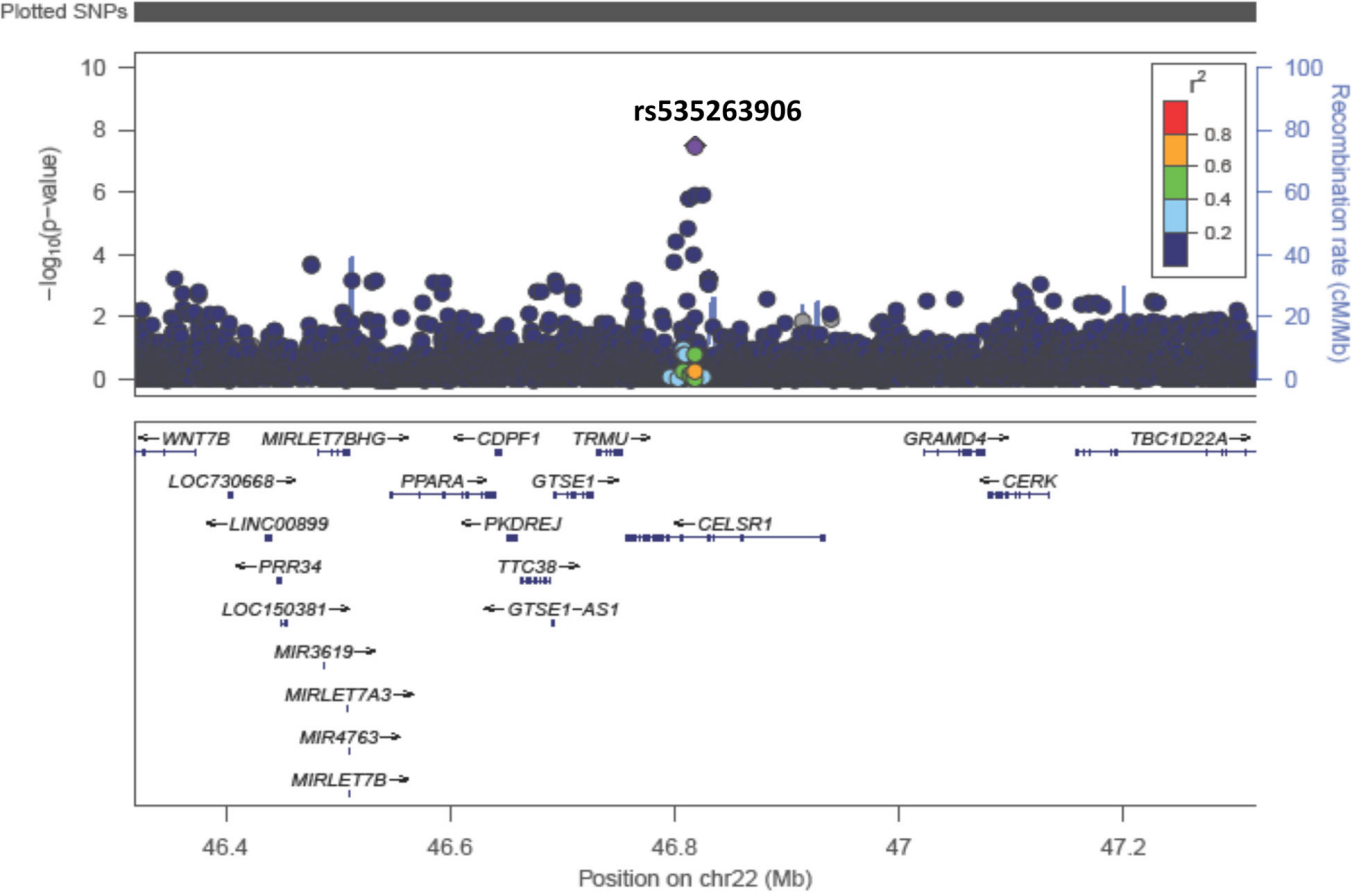
1 MB view of statistically significant peak on chromosome 22 with gene tracks overlaid

The analysis was adjusted for self-identified race (African American or white) and the analytic method accounted for population stratification and relatedness of individuals (i.e., kinship). These primary results (model 1) are depicted in the Manhattan plot in Fig. 1 (Q-Q plot depicted in Fig. 2). The top 20 SNPs associated with heart rate are listed in Table 3, showing the allele frequencies and effect sizes, and whether the SNP was present in only one racial group or not. One locus, rs535263906 on chromosome 22q13, met the genome-wide statistical significance (*p* = 3.3 × 10^−8^). A zoomed in view (1 Mb) of this association peak is shown in Fig. 3. This association peak lies within the gene Cadherin EGF LAG Seven-Pass G-Type Receptor 1 (*CELSR1)*, though there are a number of other genes nearby.

**Table 3:** Top 20 GWAS results from model 1: SNP + race + kinship (*n* = 1043; AA = 520, white = 523)

SNP	A1	A2	MAF_AA	MAF_W	MAF_All	Coef	*P* value	Group
rs535263906	A	G	0.02381	NA	0.02381	14.768	3.30E−08	AA only
rs149447933	G	C	0.01524	NA	NA	17.793	7.51E−08	AA only
rs541284506	A	G	0.01714	NA	NA	17.652	9.52E−08	AA only
rs11006544	C	T	0.01714	NA	NA	16.175	2.27E−07	AA only
rs112434206	G	A	0.02286	NA	NA	14.598	2.77E−07	AA only
rs11110004	C	T	0.01143	NA	NA	19.543	2.97E−07	AA only
rs114821210	A	C	0.01714	NA	NA	15.695	5.30E−07	AA only
rs74056623	A	G	0.05238	NA	NA	9.270	6.46E−07	AA only
rs148133894	C	T	0.00952	NA	NA	20.479	8.64E−07	AA only
rs189919070	T	C	0.00762	NA	NA	22.822	9.33E−07	AA only
rs74864598	A	C	0.09524	0.01912	0.05725	5.793	1.01E−06	Complete
rs16917667	A	G	0.09524	0.01912	0.05725	5.793	1.01E−06	Complete
rs74056624	A	G	0.04952	NA	NA	9.318	1.06E−06	AA only
rs149322277	T	C	0.02286	NA	NA	13.281	1.14E−06	AA only
rs188344082	A	G	0.04952	NA	NA	9.287	1.16E−06	AA only
rs150381023	C	T	0.02	NA	NA	14.152	1.21E−06	AA only
rs150109621	T	C	0.02	NA	NA	14.152	1.21E−06	AA only
rs8105292	C	T	0.3371	0.1128	0.2252	3.433	1.28E−06	Complete
rs139130723	G	A	0.01714	NA	NA	15.126	1.33E−06	AA only
rs142803096	C	G	0.01714	NA	0.01714	15.126	1.33E−06	AA only

There were two additional association peaks that were close to statistical significance in the primary analysis. One was on chromosome 5 centered on rs541284506 (*P* = 9.5 × 10^−8^), and another was on chromosome 6 with the strongest SNP being rs149447933 (*P* = 7.5 × 10^−8^). These peaks were somewhat broad, did not have clear supporting base of SNPs, and did not locate within a known coding gene; the closest being roughly 200 kb from s149447933 on Chr6 (a non-coding RNA gene, *LINC01108*). Close up (1 Mb) images of these two peaks are shown in Additional file 2: Figure S1 and Additional file 3: Figure S2. There were an additional 138 SNPs with levels of association considered suggestive (*P* < 10^−5^), a full listing of which are included in Additional file 1: Table S2. Finally, we stratified model 1 by race (Additional file 1: Tables S6 and S7).

### Sensitivity and other additional analyses

**Fig. 4:**
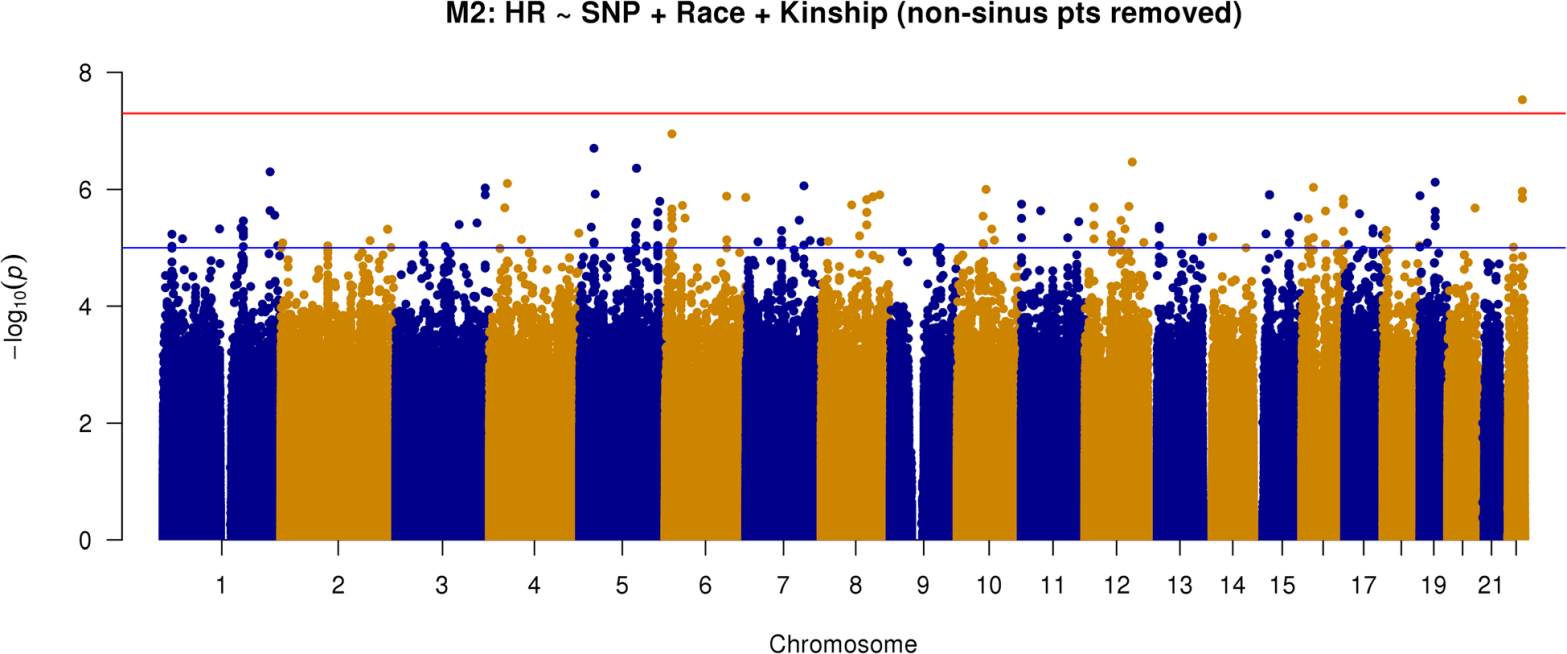
Manhattan plot of GWAS results from model 2: SNP + race + kinship; excluding possible non-sinus patients (*n* = 901; AA = 484, white = 417). Blue reference line 1 × 10^−5^; red reference line 5 × 10^−8^

**Fig. 5:**
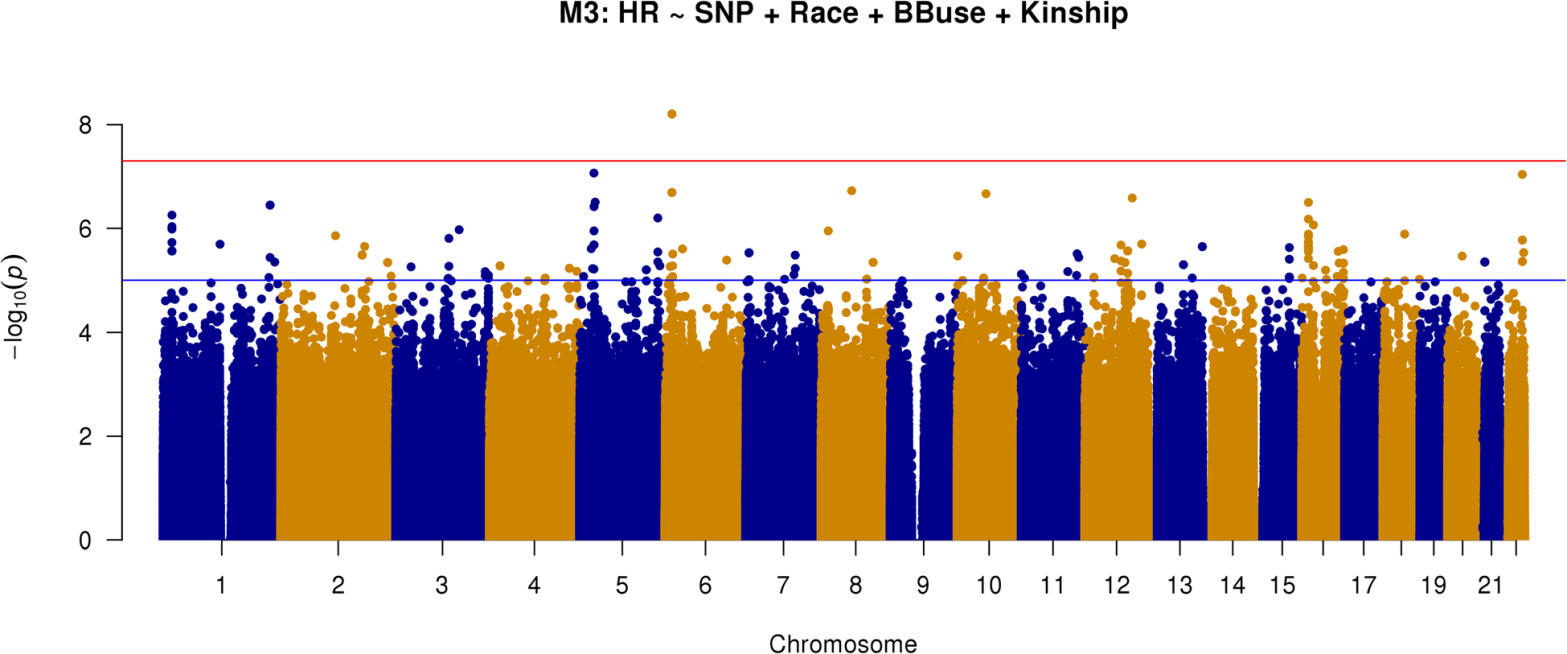
Manhattan plot of GWAS results from model 3: SNP + race + kinship + BB exposure (*n* = 990; AA = 494, white = 496). Blue reference line 1 × 10^−5^; red reference line 5 × 10^−8^

We performed several additional genome wide analyses to mitigate any contributions from possible confounding factors and assess the robustness of the above findings. First, we performed a subgroup analysis excluding patients classified as possible non- sinus rhythm. Because ECG was not obtained as part of study enrollment and thus could not guarantee sinus rhythm in all subjects, we performed additional investigations collecting and examining all clinically available ECG in patients with any prior diagnosis of atrial arrhythmia. These patients were classified as possible non-sinus or likely sinus based on ECG evidence of heart rhythm near the time of enrollment. We then repeated our primary statistical analysis excluding patients deemed as possible non-sinus rhythm. This analysis (model 2) has results shown in Fig. 4 and the top 20 loci listed in Table 4. Another key secondary analysis was similar to the primary analysis (i.e., all subjects) but adjusted for beta-blocker exposure (model 3). These results are depicted in Fig. 5 and top loci listed in Table 5. Overall, the results of models 2 and 3 appear similar to the results of the primary analysis (model 1). We still see the same three loci of potential interest though there is variation in terms of priority and whether they meet genome-wide statistical significance. Specifically, the most strongly associated SNP in model 1 was still so for model 2 (rs541284506) but was third in model 3, achieving *P* = 9.97 × 10^−8^), while rs149447933 achieved statistical significance in this model (*P* = 6.29 × 10^−9^).

**Table 4:** Top 20 GWAS results from model 2: SNP + race + kinship; excluding possible non-sinus patients (*n* = 901; AA = 484, white = 417)

SNP	A1	A2	MAF_AA	MAF_W	MAF_All	Coef	*P* value	Group
rs535263906	A	G	0.02381	NA	NA	15.372	2.94E−08	AA only
rs149447933	G	C	0.01524	NA	NA	18.040	1.12E−07	AA only
rs541284506	A	G	0.01714	NA	NA	18.282	1.97E−07	AA only
rs11110004	C	T	0.01143	NA	NA	19.375	3.39E−07	AA only
rs79031501	C	T	NA	0.02294	NA	14.281	4.33E−07	EA only
rs965460	G	A	NA	0.02294	NA	14.281	4.33E−07	EA only
rs114726259	T	C	NA	0.02199	NA	14.281	4.33E−07	EA only
rs148467525	A	G	0.01429	NA	NA	18.407	4.99E−07	AA only
rs8105292	C	T	0.3371	0.1128	0.2252	3.687	7.52E−07	Complete
rs78829380	T	C	0.06571	NA	NA	8.326	7.89E−07	AA only
rs111681691	T	C	0.02381	0.0392	0.03149	8.908	8.67E−07	Complete
rs148133894	C	T	0.00952	NA	NA	20.344	9.22E−07	AA only
rs189566544	C	A	0.0219	NA	NA	14.624	9.43E−07	AA only
rs11006544	C	T	0.01714	NA	NA	15.681	9.98E−07	AA only
rs150381023	C	T	0.02	NA	NA	14.870	1.08E−06	AA only
rs150109621	T	C	0.02	NA	NA	14.870	1.08E−06	AA only
rs112434206	G	A	0.02286	NA	NA	14.067	1.20E−06	AA only
rs117130066	A	G	NA	0.02677	NA	12.323	1.23E−06	EA only
rs74866062	C	A	NA	0.01625	NA	16.806	1.24E−06	EA only
rs117289820	C	G	NA	0.01625	0.01625	16.806	1.24E−06	EA only

**Table 5:** Top 20 GWAS results from model 3: SNP + race + kinship + BB exposure (*n* = 990; AA = 494, white = 496)

SNP	A1	A2	MAF_AA	MAF_EA	MAF_All	Coef	*P* value	Group
rs149447933	G	C	0.01524	NA	NA	19.714	6.29E−09	AA only
rs541284506	A	G	0.01714	NA	NA	17.634	8.59E−08	AA only
rs535263906	A	G	0.02381	NA	NA	14.516	9.12E−08	AA only
rs187251765	A	C	0.01333	NA	NA	19.092	1.88E−07	AA only
rs139130723	G	A	0.01714	NA	NA	16.629	2.04E−07	AA only
rs142803096	C	G	0.01714	NA	NA	16.629	2.04E−07	AA only
rs11006544	C	T	0.01714	NA	NA	16.148	2.15E−07	AA only
rs11110004	C	T	0.01143	NA	NA	19.544	2.60E−07	AA only
rs112434206	G	A	0.02286	NA	NA	14.507	3.12E−07	AA only
rs6498482	T	C	0.3752	0.588	0.4814	− 2.987	3.16E−07	Complete
rs143554223	G	A	0.01333	NA	NA	19.333	3.56E−07	AA only
rs148467525	A	G	0.01333	NA	NA	19.333	3.56E−07	AA only
rs188482801	A	C	0.03238	NA	NA	12.604	3.81E−07	AA only
rs74056623	A	G	0.05238	NA	NA	9.378	5.55E−07	AA only
rs114821210	A	C	0.01714	NA	NA	15.541	6.32E−07	AA only
rs7188980	C	T	0.4143	0.6195	0.5167	2.859	6.63E−07	Complete
rs148133894	C	T	0.00952	NA	NA	20.392	8.60E−07	AA only
rs74056624	A	G	0.04952	NA	NA	9.350	9.24E−07	AA only
rs188344082	A	G	0.04952	NA	NA	9.389	1.03E−06	AA only
rs189919070	T	C	0.00762	NA	NA	22.630	1.06E−06	AA only

We also performed an additional subgroup analysis restricted to patients with EF ≤ 35% (*n* = 589). The analysis of this much smaller group yielded no variants that met whole genome significance. There was one locus with two SNPs in proximity to each other, rs76008716 and rs57957360, which were near statistical significance (*P* = 5.4 × 10^−8^) and had not appeared in the top loci of other analyses. We performed a sensitivity analysis treating HR as an ordinal variable (rather than continuous variable), thereby mitigating the influence of very low or very high values and not overestimating precision of measure. These results were overall similar to the above with two of the top three SNPs being the same (Additional file 1: Table S5).

### Gene-wise analysis of heart rate

As an alternative approach to try to identify genes impacting heart rate, we performed another genome-wide analysis testing gene regions rather than individual SNPs. A total of 42,774 genes/functional genomic regions were defined based upon the ENCODE project annotation. The critical *P* value for global error rate was 1.17 × 10^−6^, and overall results are depicted in Additional file 4: Figure S3 (Q-Q plot depicted in Additional file 5: Figure S4). No genes met genome-wide significance. The top 40 genes, which had *P* < 0.001, are shown in Additional file 1: Table S3. There were four genes with *P* values < 1 × 10^−4^. All four of these gene regions are currently of unknown function (Additional file 1: Table S4). One gene on chromosome 12 (C12orf74) was predicted to be a protein-encoding gene, though its structure and function remain unknown. The other three gene-regions of interest were in non-coding RNA genes, also of unknown function.

## Discussion

Although similar types of studies have been performed previously in population samples [10–13], our study is the first that we know of to attempt to identify genomic regions that influence HR in stable patients with HFrEF. Analysis of this diverse patient cohort revealed a modest but statistically significant difference in HR between self-identifying white and AAs (with AA showing a slightly higher HR) as well as three genomic loci at or near genome-wide significance in at least one analysis for association to HR, of particular interest is a novel locus on Chr. 22q13 in the gene *CELSR1*.

Putting this study in context of pre-existing work, we were able to replicate one of the previously published genetic loci, in the gene *GJA1*, which had been reported to be associated with resting HR in multiple studies. This not only reconfirms the validity of this association but also suggests that *GJA1* genetic variation may have impact on HR in patients with HFrEF. High resting heart rate is a well-recognized modifiable risk factor for cardiovascular morbidity and mortality in heart failure patients [3–6]. GJA1 encodes connexin 43, a connexin family protein and a major component of the cardiac gap junction which is a central in the electrical coupling of cardiac myocytes [22]. The fact that we did not replicate the other GW significant hits from previous studies could be due in part to the relatively smaller size of our cohort, but importantly, there are design differences that may also contribute to differential findings. Most important is that this cohort was all the HFrEF patients, and the genetic factors influencing HR may be different in the setting of this disease. This is in fact the primary reason we performed this analysis, to attempt to identify HR mechanism relevant to HF that may thus impact therapy or patient outcomes. Another important difference is the fact that our cohort is racially diverse while most previous studies were in only patients of European ancestry. This diverse cohort allows us to potentially detect important associations that may be amplified or selective to AA patients and is another potential reason for differential finding in ours vs. previous studies. The strongest associated loci are in fact specific to African American patients in this cohort.

The SNP-wise GWA analysis identified two SNPs that met whole genome significance for association with HR in one or more models (see Tables 3–5) and another that was very close in each analysis. The strongest association appeared to be for the association peak on Chr 22. The peak SNP in this analysis (rs535263906) was only present and tested in African American patient samples, and while imputed (rather than directly genotyped), it was of high imputation confidence (*R*^2^ = 0.816) and had a supporting peak of SNPs beneath it. This association peak is near a large number of genes but lies almost completely within *CELSR1*, which encodes a receptor of the cadherin super-family and appears to have gene expression (mRNA) in a wide range of tissues including in cardiac and smooth muscle cells (The Genotype-Tissue Expression [GTEx] project). The association peak encompasses most of the 5′ half of *CELSR1*, so attempting to infer possible functional impact of the yet unidentified causative variant (presumably linked to rs535263906 but accounting for the phenotype association) would be speculative and remains the work of future investigation. Interestingly, *CELSR1* has been reported in other GWAS studies as being associated with a variety of traits; these include stroke [23] and a suggestive association with fenofibrate response in diabetics [24]. Further investigation into this gene and the other candidates is needed to assess their possible cardiac functional impact. While whole-genome significance was met, these findings should be viewed as preliminary until they can be tested for validation in another data set. Unfortunately, another similar data set, particularly including African Americans with HFrEF is not readily available to the investigative team.

Although no statistical significance was reached in the gene-wise analysis, these results can be viewed as a prioritization list for possible association to HR among HF patients with HFrEF. Similarly, the many additional individual SNPs that were in the range of possible interest for association with HR (i.e., having *P* values < 10^−6^.) may be of value for future investigation. A high proportion of these loci seemed specific to AA. This could be due to a higher number of SNPs in AA, statistical chance, or perhaps because HR is under a stronger genetic influence in this group of patients. The analysis of very low EF (< 35%) yielded two additional SNPs of interests, rs76008716 and rs57957360, which were very near statistical significance (*P* = 5.4 × 10^−8^). These loci are located in an intergenic region on chromosome 5 and are of unclear biological significance at this point. The results of this much smaller subcohort should be interpreted with caution until validated externally.

This study has several limitations. First, it is derived from a single center cohort of modest size for a GWAS. Mitigating this is that we are modeling a continuous variable as endpoint which provides more statistical power compared to dichotomous or event-driven analyses. In terms of phenotype, ECG was not performed on the day of enrollment in our study, and pulse rate was used as our HR measure. For most patients, pulse rate should be a reliable measure of HR but this may have differences compared to ECG-derived measures. Moreover, the lack of an ECG makes it impossible for us to state with certainty that subjects were in sinus rhythm at the time of assessment, but we have worked hard to mitigate this risk, including additional analyses excluding patients with any history of atrial arrhythmia. Another potential concern is that we used EF < 50% as enrollment criteria to categorize patients as HFrEF. While this is no longer the standard, it was so at the time of the study initiation and we performed additional analyses to assess any potential impact. Finally, while we adjusted for beta-blocker exposure, we did not have an actual level the day of enrollment. Despite this, the medication exposure metric used has been previously shown to correlate to clinical outcomes and inversely correlate with HR [25] and thus is likely to be a good estimate of true exposure.

## Conclusions

In summary, HR in the setting of HFrEF appears to be impacted by genetic factors. One previous candidate gene was confirmed in the setting of HFrEF (*GJA1*), and GWAS of HR identified several novel genomic loci associated with HR, particularly a statistically significant peak in the gene *CELSR1*. These novel candidate genes for HR in the setting of HFrEF require additional investigation and validation.

## Additional files

Additional file 1: **Table S1.** Loci from of other GWAS studies tested in HFrEF patients. **Table S2.** Additional GWAS results of potential interest *(P* < 10^− 5^) from model 1 (*n* = 1043; AA = 520, white = 523). **Table S3.** Association of genes with heart rate in EF patients (from gene-wise analysis). **Table S4.** Classification of genes of interest from gene-wise analysis. **Table S5.** Sensitivity analysis of HR an ordinal variable, *n* = 1043 (AA 520, EA 523). **Table S6.** Top 20 SNPs from 520 AA patients: model 1 (SNP + race + kinship). **Table S7.** Top 20 SNPs from 523 EA patients: model 1 (SNP + race + kinship) (DOCX 59 kb)

Additional file 2: **Figure S1.** Close up (1 Mb) Manhattan plot of Chr. 5 association peak (TIF 14502 kb)

Additional file 3: **Figure S2.** Close up (1 Mb) Manhattan plot of Chr. 6 association peak (TIF 2869 kb)

Additional file 4: **Figure S3.** Manhattan Plot of the gene-wise analysis of heart rate. Blue reference line: 1x10^-5^, Red reference line: 1x10^-4^. (TIFF 14100 kb)

Additional file 5: **Figure S4.** Q-Q Plot of SKAT P values for approximately 43,000 genes from the gene-wise analysis of heart rate. (TIFF 16400 kb)

## Abbreviations

AA: African American
BB: Beta-blocker
BPM: Beats per minute
COPD: Chronic obstructive pulmonary disease
ECG: Electrocardiogram
EF: Ejection fraction
EMR: Electronic medical record
GenHRate: Genetics of Heart Rate Observational Study
HAP: Health Alliance Plan
HFMG: Henry Ford Medical Group.
HF: Heart failure
HFHS: Henry Ford Health System
HFrEF: Heart failure with reduced ejection fraction
HR: Heart rate
SNP: Single nucleotide polymorphisms
