# Supplementary material for: Genetics of heart rate in heart failure patients (GenHRate)
Source: Hum Genomics. 2019 May 21;13:22. doi: 10.1186/s40246-019-0206-6 (PMC6528282; doi:10.1186/s40246-019-0206-6)
Supplement: Supplementary file 1 — Table S1. Loci from of other GWAS studies tested in HFrEF patients. Table S2. Additional GWAS results of potential interest (P < 10− 5) from model 1 (n = 1043; AA = 520, white = 523). Table S3. Association of genes with heart rate in EF patients (from gene-wise analysis). Table S4. Classification of genes of interest from gene-wise analysis. Table S5. Sensitivity analysis of HR an ordinal variable, n = 1043 (AA 520, EA 523). Table S6. Top 20 SNPs from 520 AA patients: model 1 (SNP + race + kinship). Table S7. Top 20 SNPs from 523 EA patients: model 1 (SNP + race + kinship) (DOCX 59 kb) [file 40246_2019_206_MOESM1_ESM.docx]

**Additional file 1**

**Table S1.** Loci from of other GWAS studies tested in HFrEF patients.

| SNP | Location | SNP | Location |
| --- | --- | --- | --- |
| rs11118555 | 1:207940853 | **rs314370** | 7:100453208 |
| rs12731740 | 1:208024820 | **rs12666989** | 7:100486754 |
| rs2745967 | 1:208128722 | **rs13245899** | 7:100497131 |
| rs17853159 | 1:45810865 | **rs2350782** | 7:136642634 |
| rs709209 | 1:6278414 | **rs1635852** | 7:28189411 |
| rs17362588 | 2:179721046 | **rs180242** | 7:93549596 |
| rs4140885 | 2:188333064 | **rs3793706** | 10:102269085 |
| rs13030174 | 2:232271284 | **rs10857472** | 10:50534599 |
| rs9647379 | 3:171785168 | **rs174547** | 11:61570783 |
| rs7612445 | 3:179172979 | **rs174549** | 11:61571382 |
| rs6795970 | 3:38766675 | **rs2067615** | 12:107149422 |
| rs3087866 | 3:49054692 | **rs885389** | 12:131621762 |
| rs6882776 | 5:172664163 | **rs17287293** | 12:24770878 |
| rs6893300 | 5:179135815 | **rs7980799** | 12:33576990 |
| rs4282331 | 5:30881510 | **rs826838** | 12:39106731 |
| rs281868 | 6:118574061 | **rs365990** | 14:23861811 |
| rs11153730 | 6:118667522 | **rs452036** | 14:23865885 |
| rs11154022 | 6:121748542 | **rs223116** | 14:23977010 |
| rs9320841 | 6:122114451 | **rs17796783** | 14:85809911 |
| rs1015451 | 6:122131485 | **rs6127471** | 20:36844038 |
| rs9398652 | 6:122146034 | **rs12004** | 22:38877461 |
| rs12110693 | 6:122158270 |  |  |

**Table S2.** Additional GWAS results of potential interest (p < 10^-5^) from Model 1 (n = 1043; AA=520, white =523)

| SNP | A1 | A2 | MAF_AA | MAF_EA | MAF_All | Coef | P-value | Group |
| --- | --- | --- | --- | --- | --- | --- | --- | --- |
| rs144303414 | C | A | NA | 0.02581 | 0.02581 | 11.885 | 1.48E-06 | EA only |
| rs9470398 | C | T | 0.1248 | NA | 0.1248 | -5.807 | 1.50E-06 | AA only |
| rs190258023 | A | G | 0.02 | NA | 0.02 | 13.990 | 1.53E-06 | AA only |
| rs148416395 | C | T | 0.02 | NA | 0.02 | 13.990 | 1.53E-06 | AA only |
| rs187251765 | A | C | 0.01333 | NA | 0.01333 | 17.045 | 1.55E-06 | AA only |
| rs144322502 | T | G | 0.01905 | NA | 0.01905 | 14.277 | 1.67E-06 | AA only |
| rs6498482 | T | C | 0.3752 | 0.588 | 0.4814 | -2.717 | 1.91E-06 | Complete |
| rs371848093 | C | T | 0.01143 | NA | 0.01143 | 19.805 | 1.99E-06 | AA only |
| rs78133413 | A | G | NA | 0.01816 | 0.01816 | 13.857 | 2.05E-06 | EA only |
| rs113459855 | T | A | 0.05048 | NA | 0.05048 | 8.997 | 2.05E-06 | AA only |
| rs115146744 | C | T | 0.01714 | NA | 0.01714 | 14.053 | 2.18E-06 | AA only |
| rs114101629 | C | T | 0.01714 | NA | 0.01714 | 14.797 | 2.32E-06 | AA only |
| rs79272715 | A | C | 0.2105 | 0.03728 | 0.124 | 4.196 | 2.38E-06 | Complete |
| rs143554223 | G | A | 0.01333 | NA | 0.01333 | 16.712 | 2.43E-06 | AA only |
| rs148467525 | A | G | 0.01333 | NA | 0.01333 | 16.712 | 2.43E-06 | AA only |
| rs1392797 | G | C | 0.1 | 0.01912 | 0.05964 | 5.467 | 2.50E-06 | Complete |
| rs78897914 | G | A | 0.1 | 0.01912 | 0.05964 | 5.455 | 2.63E-06 | Complete |
| rs16917715 | G | A | 0.1 | 0.01912 | 0.05964 | 5.455 | 2.63E-06 | Complete |
| rs28523422 | T | G | 0.01143 | NA | 0.01143 | 17.866 | 2.76E-06 | AA only |
| rs74056619 | A | G | 0.05238 | NA | 0.05238 | 8.702 | 3.02E-06 | AA only |
| rs74056620 | T | A | 0.05238 | NA | 0.05238 | 8.702 | 3.02E-06 | AA only |
| rs74056621 | A | C | 0.05238 | NA | 0.05238 | 8.702 | 3.02E-06 | AA only |
| rs74056622 | G | A | 0.05238 | NA | 0.05238 | 8.702 | 3.02E-06 | AA only |
| rs115982993 | T | C | 0.01238 | NA | 0.01238 | 17.098 | 3.17E-06 | AA only |
| rs111657631 | T | G | 0.01048 | NA | 0.01048 | 18.532 | 3.21E-06 | AA only |
| rs113737900 | A | G | NA | 0.02103 | 0.02103 | 12.714 | 3.24E-06 | EA only |
| rs189566544 | C | A | 0.0219 | NA | 0.0219 | 13.299 | 3.27E-06 | AA only |
| rs143694932 | A | G | 0.01048 | NA | 0.01048 | 17.015 | 3.32E-06 | AA only |
| rs75729550 | T | C | 0.01619 | NA | 0.01619 | 14.945 | 3.49E-06 | AA only |
| rs138005219 | T | C | 0.01429 | NA | 0.01429 | 15.878 | 3.68E-06 | AA only |
| rs77506079 | A | C | NA | 0.02581 | 0.02581 | 11.399 | 3.93E-06 | EA only |
| rs144563425 | G | A | 0.02286 | NA | 0.02286 | 12.914 | 3.94E-06 | AA only |
| rs146219909 | G | A | 0.01524 | NA | 0.01524 | 15.349 | 4.05E-06 | AA only |
| rs74972015 | T | C | 0.01524 | NA | 0.01524 | 15.301 | 4.07E-06 | AA only |
| rs76345468 | T | C | 0.01524 | NA | 0.01524 | 15.301 | 4.07E-06 | AA only |
| rs75262741 | A | G | 0.01524 | NA | 0.01524 | 15.301 | 4.07E-06 | AA only |
| rs147545594 | C | A | 0.01524 | NA | 0.01524 | 15.301 | 4.07E-06 | AA only |
| rs116454494 | G | C | 0.101 | 0.01912 | 0.06011 | 5.335 | 4.15E-06 | Complete |
| rs7188980 | C | T | 0.4143 | 0.6195 | 0.5167 | 2.583 | 4.18E-06 | Complete |
| rs189536067 | A | G | 0.01619 | NA | 0.01619 | 14.753 | 4.21E-06 | AA only |
| [rs111641830](http://www.ncbi.nlm.nih.gov/SNP/snp_ref.cgi?type=rs&rs=rs111641830) | T | C | 0.01619 | NA | 0.01619 | 14.753 | 4.21E-06 | AA only |
| [rs112372754](http://www.ncbi.nlm.nih.gov/SNP/snp_ref.cgi?type=rs&rs=rs112372754) | T | C | 0.01619 | NA | 0.01619 | 14.753 | 4.21E-06 | AA only |
| rs7198756 | G | A | 0.4667 | 0.4025 | 0.4346 | 2.615 | 4.46E-06 | Complete |
| rs142659860 | A | G | 0.01143 | NA | 0.01143 | 19.160 | 4.51E-06 | AA only |
| rs73059342 | C | T | 0.01143 | NA | 0.01143 | 19.160 | 4.51E-06 | AA only |
| [rs556723179](http://www.ncbi.nlm.nih.gov/SNP/snp_ref.cgi?type=rs&rs=rs556723179) | C | T | 0.01143 | NA | 0.01143 | 19.160 | 4.51E-06 | AA only |
| rs76806081 | T | C | 0.02286 | NA | 0.02286 | 12.006 | 4.58E-06 | AA only |
| rs587606498 | T | C | 0.05143 | 0.02008 | 0.03578 | 7.086 | 4.64E-06 | Complete |
| rs5752592 | A | G | 0.08381 | 0.1224 | 0.1031 | 4.127 | 4.68E-06 | Complete |
| rs28580426 | C | A | 0.04952 | 0.1224 | 0.08588 | 4.508 | 4.72E-06 | Complete |
| rs76392993 | T | C | NA | 0.01147 | 0.01147 | 16.699 | 4.78E-06 | EA only |
| rs114782882 | A | C | 0.01905 | NA | 0.01905 | 12.963 | 4.90E-06 | AA only |
| [rs552214848](http://www.ncbi.nlm.nih.gov/SNP/snp_ref.cgi?type=rs&rs=rs552214848) | A | T | 0.1133 | NA | 0.1133 | 5.770 | 4.93E-06 | AA only |
| rs6009185 | A | G | 0.4905 | 0.3193 | 0.4051 | 2.668 | 5.09E-06 | Complete |
| rs372344 | C | T | 0.3429 | 0.5545 | 0.4485 | -2.610 | 5.09E-06 | Complete |
| rs17757727 | T | G | NA | 0.01721 | 0.01721 | 13.681 | 5.14E-06 | EA only |
| rs139489372 | A | C | NA | 0.01721 | 0.01721 | 13.675 | 5.15E-06 | EA only |
| rs114512805 | T | C | 0.01524 | NA | 0.01524 | 15.160 | 5.15E-06 | AA only |
| rs139731147 | A | G | 0.02667 | NA | 0.02667 | 11.566 | 5.19E-06 | AA only |
| rs116598880 | T | C | 0.02762 | NA | 0.02762 | 11.352 | 5.20E-06 | AA only |
| rs10234809 | C | T | 0.2333 | 0.3795 | 0.3063 | 2.796 | 5.27E-06 | Complete |
| rs76302892 | A | C | 0.01333 | NA | 0.01333 | 16.109 | 5.41E-06 | AA only |
| rs111681691 | T | C | 0.02381 | 0.0392 | 0.03149 | 7.252 | 5.49E-06 | Complete |
| rs79031501 | C | T | NA | 0.02294 | 0.02294 | 11.401 | 5.62E-06 | EA only |
| rs965460 | G | A | NA | 0.02294 | 0.02294 | 11.401 | 5.62E-06 | EA only |
| rs113516553 | T | G | 0.03619 | NA | 0.03619 | 10.020 | 5.84E-06 | AA only |
| rs113983785 | G | C | 0.03619 | NA | 0.03619 | 10.020 | 5.84E-06 | AA only |
| rs111371067 | A | G | 0.03619 | NA | 0.03619 | 10.020 | 5.84E-06 | AA only |
| rs138517179 | T | C | 0.03619 | NA | 0.03619 | 10.020 | 5.84E-06 | AA only |
| rs146264611 | C | T | 0.01905 | NA | 0.01905 | 12.864 | 5.86E-06 | AA only |
| rs114726259 | T | C | NA | 0.02199 | 0.02199 | 11.591 | 5.89E-06 | EA only |
| rs116116894 | T | C | 0.02571 | NA | 0.02571 | 11.249 | 5.95E-06 | AA only |
| rs149649230 | T | C | 0.02571 | NA | 0.02571 | 11.250 | 6.24E-06 | AA only |
| rs143054558 | C | T | NA | 0.01816 | 0.01816 | 13.207 | 6.29E-06 | EA only |
| rs8181669 | C | T | 0.4286 | 0.7964 | 0.6121 | -2.824 | 6.31E-06 | Complete |
| rs1490716 | A | G | 0.4295 | 0.7964 | 0.6126 | -2.820 | 6.39E-06 | Complete |
| rs112901026 | G | T | 0.01429 | NA | 0.01429 | 15.472 | 6.41E-06 | AA only |
| rs34141129 | A | T | 0.4562 | 0.4503 | 0.4532 | 2.610 | 6.42E-06 | Complete |
| rs79087352 | C | A | 0.01048 | NA | 0.01048 | 17.931 | 6.47E-06 | AA only |
| rs78829380 | T | C | 0.06571 | NA | 0.06571 | 7.342 | 6.55E-06 | AA only |
| rs58829444 | C | T | 0.2533 | 0.2266 | 0.24 | 2.956 | 6.74E-06 | Complete |
| rs62509389 | C | T | NA | 0.01147 | 0.01147 | 16.443 | 6.74E-06 | EA only |
| rs62509394 | C | T | NA | 0.01147 | 0.01147 | 16.443 | 6.74E-06 | EA only |
| rs149765481 | A | C | 0.0181 | NA | 0.0181 | 13.046 | 6.86E-06 | AA only |
| rs13338660 | T | G | 0.3533 | 0.5793 | 0.4661 | -2.562 | 6.98E-06 | Complete |
| rs190116644 | T | C | NA | 0.008604 | 0.008604 | 18.861 | 7.08E-06 | EA only |
| rs182178320 | T | C | 0.0219 | NA | 0.0219 | -12.519 | 7.18E-06 | AA only |
| rs192154334 | C | A | 0.01333 | NA | 0.01333 | -15.899 | 7.21E-06 | AA only |
| rs115709306 | T | C | 0.01238 | NA | 0.01238 | 16.459 | 7.41E-06 | AA only |
| rs78705027 | C | A | 0.01238 | NA | 0.01238 | 16.459 | 7.41E-06 | AA only |
| rs116175387 | T | C | 0.01238 | NA | 0.01238 | 16.459 | 7.41E-06 | AA only |
| rs115472750 | A | G | 0.02 | NA | 0.02 | 13.012 | 7.63E-06 | AA only |
| rs116213227 | A | G | 0.02857 | NA | 0.02857 | 11.008 | 7.70E-06 | AA only |
| rs9924452 | T | C | 0.4143 | 0.6185 | 0.5162 | 2.508 | 7.71E-06 | Complete |
| rs188748322 | C | T | 0.01619 | NA | 0.01619 | 14.415 | 7.74E-06 | AA only |
| rs7184192 | A | C | 0.3724 | 0.5803 | 0.4761 | -2.536 | 7.75E-06 | Complete |
| rs72671743 | G | A | 0.02286 | 0.08891 | 0.05582 | 5.409 | 7.81E-06 | Complete |
| rs114700275 | T | C | 0.02952 | NA | 0.02952 | 10.825 | 7.95E-06 | AA only |
| rs114908471 | A | G | 0.02952 | NA | 0.02952 | 10.825 | 7.95E-06 | AA only |
| rs11766034 | T | C | NA | 0.01052 | 0.01052 | 16.992 | 7.96E-06 | EA only |
| rs1477717 | T | C | 0.2286 | 0.02581 | 0.1274 | 3.958 | 8.12E-06 | Complete |
| rs17097649 | A | G | 0.008571 | NA | 0.008571 | 19.527 | 8.32E-06 | AA only |
| rs17097676 | G | T | 0.008571 | NA | 0.008571 | 19.527 | 8.32E-06 | AA only |
| rs113510721 | T | C | 0.02095 | NA | 0.02095 | 12.699 | 8.44E-06 | AA only |
| rs28806579 | A | G | 0.02095 | NA | 0.02095 | 12.699 | 8.44E-06 | AA only |
| rs116521297 | T | C | 0.05714 | NA | 0.05714 | 7.734 | 8.49E-06 | AA only |
| rs78093250 | A | G | 0.05714 | NA | 0.05714 | 7.734 | 8.49E-06 | AA only |
| rs138575291 | C | T | 0.02286 | NA | 0.02286 | 12.155 | 8.65E-06 | AA only |
| rs74956835 | A | C | 0.01524 | NA | 0.01524 | 14.755 | 8.71E-06 | AA only |
| rs1364363 | C | G | 0.3543 | 0.586 | 0.4699 | -2.542 | 8.92E-06 | Complete |
| rs1678921 | C | T | 0.3371 | 0.5535 | 0.4451 | -2.538 | 8.97E-06 | Complete |
| rs77095672 | T | C | 0.2019 | NA | 0.2019 | 4.294 | 9.12E-06 | AA only |
| rs56032548 | A | G | 0.2019 | NA | 0.2019 | 4.294 | 9.12E-06 | AA only |
| rs76064792 | A | G | 0.01524 | NA | 0.01524 | 14.724 | 9.15E-06 | AA only |
| rs112577387 | T | C | 0.01619 | NA | 0.01619 | 14.296 | 9.20E-06 | AA only |
| rs73123536 | T | C | 0.01619 | NA | 0.01619 | 14.296 | 9.20E-06 | AA only |
| rs10163219 | A | G | 0.3552 | 0.5813 | 0.468 | -2.537 | 9.21E-06 | Complete |
| rs9927170 | T | C | 0.3581 | 0.5813 | 0.4695 | -2.525 | 9.23E-06 | Complete |
| rs2092866 | T | C | 0.239 | NA | 0.239 | 4.239 | 9.25E-06 | AA only |
| rs57346421 | C | G | 0.02381 | NA | 0.02381 | 11.877 | 9.26E-06 | AA only |
| rs55798126 | G | A | 0.02381 | NA | 0.02381 | 11.877 | 9.26E-06 | AA only |
| rs56337324 | A | G | 0.02381 | NA | 0.02381 | 11.877 | 9.26E-06 | AA only |
| rs78528733 | G | A | 0.02381 | NA | 0.02381 | 11.877 | 9.26E-06 | AA only |
| rs73894141 | A | T | 0.02381 | NA | 0.02381 | 11.877 | 9.26E-06 | AA only |
| rs73894142 | T | C | 0.02381 | NA | 0.02381 | 11.877 | 9.26E-06 | AA only |
| rs56107869 | G | C | 0.06857 | 0.1281 | 0.09828 | -4.185 | 9.39E-06 | Complete |
| rs56297497 | T | C | 0.06857 | 0.1281 | 0.09828 | -4.185 | 9.39E-06 | Complete |
| rs268429 | A | G | 0.1171 | NA | 0.1171 | 5.641 | 9.70E-06 | AA only |
| rs115974372 | A | G | NA | 0.02868 | 0.02868 | 10.059 | 9.71E-06 | EA only |
| rs72993012 | A | G | 0.0419 | 0.1836 | 0.1126 | -3.879 | 9.74E-06 | Complete |
| rs17077766 | T | C | 0.1571 | 0.01243 | 0.08492 | 4.758 | 9.76E-06 | Complete |
| rs115050692 | T | C | 0.01143 | NA | 0.01143 | 16.887 | 9.77E-06 | AA only |
| rs146203779 | G | T | 0.01143 | NA | 0.01143 | 16.887 | 9.77E-06 | AA only |
| rs76882026 | A | G | 0.3324 | 0.0325 | 0.1827 | 3.599 | 9.77E-06 | Complete |
| rs186722751 | A | C | 0.009524 | NA | 0.009524 | 18.413 | 9.94E-06 | AA only |
| rs148470800 | G | T | 0.009524 | NA | 0.009524 | 18.413 | 9.94E-06 | AA only |
| rs79713328 | C | T | 0.01429 | NA | 0.01429 | 15.143 | 9.96E-06 | AA only |
| rs60100617 | C | T | 0.2857 | 0.2285 | 0.2572 | 2.868 | 9.98E-06 | Complete |

**Table S3. Association of genes with heart rate in EF patients (from gene-wise analysis)**

| Gene | P | #SNP  tested | Chr | Gene | P | #SNP  tested | Chr |
| --- | --- | --- | --- | --- | --- | --- | --- |
| RP11-334G22.1 | 6.47E-05 | 2 | 2 | **MIER2** | 5.77E-04 | 192 | 19 |
| RP11-499F3.2 | 6.72E-05 | 429 | 15 | **GRM4** | 6.21E-04 | 410 | 6 |
| C12orf74 | 6.96E-05 | 5 | 12 | **PIGX** | 6.24E-04 | 334 | 3 |
| AC011718.2 | 9.37E-05 | 8 | 22 | **RPS14P3** | 6.44E-04 | 3 | 1 |
| AP000295.10 | 1.27E-04 | 1 | 21 | **CHMP4B** | 6.57E-04 | 38 | 20 |
| AC093166.4 | 1.55E-04 | 1 | 2 | **BUB1B** | 6.66E-04 | 69 | 15 |
| RP11-734K21.2 | 1.58E-04 | 3 | 2 | **RP11-342M1.3** | 6.67E-04 | 14 | 1 |
| CD200R1L | 1.79E-04 | 103 | 3 | **ECEL1** | 6.85E-04 | 20 | 2 |
| RP11-36N20.1 | 2.28E-04 | 39 | 1 | **IGLJ7** | 6.88E-04 | 1 | 22 |
| RP13-539J13.1 | 2.49E-04 | 1 | 2 | **ZNF525** | 7.02E-04 | 116 | 19 |
| RP11-194G10.3 | 3.13E-04 | 4 | 3 | **RP11-430N14.4** | 7.10E-04 | 42 | 9 |
| RP3-468B3.4 | 3.14E-04 | 4 | 6 | **RP11-3D4.3** | 7.68E-04 | 6 | 15 |
| RPL3P2 | 3.39E-04 | 35 | 6 | **KCTD1** | 7.77E-04 | 508 | 18 |
| RP11-428G5.7 | 3.51E-04 | 4 | 12 | **AC093590.1** | 7.80E-04 | 48 | 2 |
| AP4B1-AS1 | 3.58E-04 | 23 | 1 | **AC003005.2** | 7.86E-04 | 15 | 19 |
| RP11-284H19.1 | 3.84E-04 | 14 | 12 | **RP11-276H7.2** | 8.28E-04 | 1 | 1 |
| MRPL36 | 3.87E-04 | 19 | 5 | **UGT2B15** | 8.33E-04 | 81 | 4 |
| AC116609.2 | 4.33E-04 | 30 | 2 | **RP11-557C18.3** | 8.70E-04 | 44 | 8 |
| RP11-793H13.10 | 4.58E-04 | 1 | 12 | **ZBTB21** | 9.30E-04 | 44 | 21 |
| XPO4 | 9.57E-04 | 241 | 13 | **BUB3** | 9.76E-04 | 15 | 10 |

**Table S4. Classification of genes of interest from gene-wise analysis**

| Gene | Function | | Source |
| --- | --- | --- | --- |
| RP11-334G22.1  (ENSG00000273063) | RNA gene  (non-coding) | https://www.ncbi.nlm.nih.gov/nuccore/551845395  http://www.genecards.org/cgi-bin/carddisp.pl?gene=ENSG00000273063&keywords=RP11-334G22,1 | |
| RP11-499F3.2  (ENSG00000259692) | RNA gene  (non-coding) | https://www.ncbi.nlm.nih.gov/nuccore/HG507323.1  http://www.genecards.org/cgi-bin/carddisp.pl?gene=ENSG00000259692&keywords=RP11-499F3,2 | |
| C12orf74 | Protein (Unknown) Coding Gene | https://www.ncbi.nlm.nih.gov/nuccore/?term=C12orf74  https://www.ncbi.nlm.nih.gov/nuccore/KJ896051.1  http://www.genecards.org/Search/Keyword?queryString=C12orf74  http://www.genecards.org/cgi-bin/carddisp.pl?gene=C12orf74&keywords=C12orf74 | |
| AC011718.2  (LOC729461) | RNA Gene, affiliated with the ncRNA class. | https://www.ncbi.nlm.nih.gov/nuccore/AC011718.2  http://www.genecards.org/cgi-bin/carddisp.pl?gene=LOC729461&keywords=AC011718,2 | |

**Table S5. Sensitivity analysis of HR an ordinal variable, n = 1043 (AA: 520, EA: 523).**

| SNP | | Location | Coef | SE | P-value |
| --- | --- | --- | --- | --- | --- |
| rs8105292 | 19:32594822 | | -0.370372892 | 0.068124229 | 6.76E-08 |
| rs535263906 | 22:46818390 | | -1.383220872 | 0.257817535 | 9.97E-08 |
| rs149447933 | 6:14454139 | | -1.693567592 | 0.318976252 | 1.34E-07 |
| rs148133894 | 16:24595999 | | -2.096728645 | 0.401008632 | 2.06E-07 |
| rs190258023 | 22:46812943 | | -1.413353805 | 0.280636536 | 5.59E-07 |
| rs148416395 | 22:46813112 | | -1.413353805 | 0.280636536 | 5.59E-07 |
| rs116598880 | 16:24512550 | | -1.1910986 | 0.240111284 | 8.20E-07 |
| rs541284506 | 5:30425529 | | -1.584131653 | 0.319503198 | 8.31E-07 |
| rs112901026 | 2:4149342 | | -1.634520248 | 0.330538252 | 8.87E-07 |
| rs74864598 | 8:96451435 | | -0.562203522 | 0.114219735 | 9.95E-07 |
| rs16917667 | 8:96451828 | | -0.562203522 | 0.114219735 | 9.95E-07 |

**Table S6. Top 20 SNPs from 520 AA patients: Model 1 (SNP + Race + Kinship)**

| SNP | Location | Coef | SE | P-value |
| --- | --- | --- | --- | --- |
| rs535263906 | 22:46818390 | -14.60322938 | 2.612359278 | 3.68E-08 |
| rs149447933 | 6:14454139 | -17.74663503 | 3.228157429 | 6.06E-08 |
| rs541284506 | 5:30425529 | -17.57919826 | 3.230924706 | 8.19E-08 |
| rs61945053 | 13:27595815 | -24.10274723 | 4.545087412 | 1.69E-07 |
| rs11006544 | 10:61270644 | -15.98006662 | 3.054764583 | 2.45E-07 |
| rs78314028 | 7:6203076 | -11.47884025 | 2.22632865 | 3.60E-07 |
| rs112434206 | 5:33083389 | -14.29215195 | 2.775457612 | 3.72E-07 |
| rs11110004 | 12:100088318 | -19.16093324 | 3.740301325 | 4.25E-07 |
| rs114821210 | 5:165516848 | -15.66652976 | 3.065606216 | 4.53E-07 |
| rs61823501 | 1:184365970 | -9.015057678 | 1.797199816 | 7.25E-07 |
| rs4233119 | 1:184341270 | -9.307108785 | 1.856090044 | 7.32E-07 |
| rs6424948 | 1:184341826 | -9.307108785 | 1.856090044 | 7.32E-07 |
| rs6424949 | 1:184341849 | -9.307108785 | 1.856090044 | 7.32E-07 |
| rs7512260 | 1:184344251 | -9.307108785 | 1.856090044 | 7.32E-07 |
| rs9470398 | 6:36767067 | 5.947437608 | 1.186969367 | 7.46E-07 |
| rs148133894 | 16:24595999 | -20.40987803 | 4.074283488 | 7.50E-07 |
| rs149322277 | 16:87958671 | -13.34535393 | 2.678485778 | 8.57E-07 |
| rs7965830 | 12:76355830 | -17.95395856 | 3.609333573 | 8.92E-07 |
| rs189919070 | 3:134155667 | -22.60711855 | 4.567157985 | 1.01E-06 |
| rs150381023 | 22:46819005 | -14.11604425 | 2.869770758 | 1.17E-06 |

**Table S7. Top 20 SNPs from 523 EA patients: Model 1 (SNP + Race + Kinship)**

| SNP | Location | Coef | SE | P-value |
| --- | --- | --- | --- | --- |
| rs1633735 | 5:8543190 | 4.150941034 | 0.833292889 | 8.60E-07 |
| rs1633734 | 5:8543334 | 4.150941034 | 0.833292889 | 8.60E-07 |
| rs1700575 | 5:8544037 | 4.150941034 | 0.833292889 | 8.60E-07 |
| rs1700576 | 5:8544182 | 4.150941034 | 0.833292889 | 8.60E-07 |
| rs1700577 | 5:8544310 | 4.150941034 | 0.833292889 | 8.60E-07 |
| rs12362161 | 11:98705232 | 4.488915802 | 0.904917369 | 9.53E-07 |
| rs403410 | 5:8543898 | 4.101979471 | 0.830398659 | 1.06E-06 |
| rs79661299 | 6:42056006 | -18.43658172 | 3.745164076 | 1.15E-06 |
| rs423297 | 5:8543442 | 4.088465468 | 0.832916454 | 1.23E-06 |
| rs418262 | 5:8544489 | 4.08491039 | 0.832591903 | 1.24E-06 |
| rs113235453 | 14:32319145 | -7.304487236 | 1.498248855 | 1.44E-06 |
| rs74633202 | 14:32324677 | -7.304487236 | 1.498248855 | 1.44E-06 |
| rs144303414 | 18:46035784 | -11.82115107 | 2.430151997 | 1.52E-06 |
| rs1709294 | 2:23750641 | -10.01645553 | 2.063593849 | 1.60E-06 |
| rs1600857 | 8:3478336 | -4.462215014 | 0.923181555 | 1.77E-06 |
| rs412078 | 5:8544779 | 3.979039995 | 0.824359361 | 1.83E-06 |
| rs78133413 | 1:236560297 | -13.84391713 | 2.874926277 | 1.93E-06 |
| rs1633733 | 5:8546006 | 4.092079982 | 0.860483116 | 2.57E-06 |
| rs1666776 | 5:8547557 | 4.092079982 | 0.860483116 | 2.57E-06 |
| rs2519719 | 5:8547970 | 4.092079982 | 0.860483116 | 2.57E-06 |
