# Supplementary figures and images for: Genetics of heart rate in heart failure patients (GenHRate)
Source: Hum Genomics. 2019 May 21;13:22. doi: 10.1186/s40246-019-0206-6 (PMC6528282; doi:10.1186/s40246-019-0206-6)

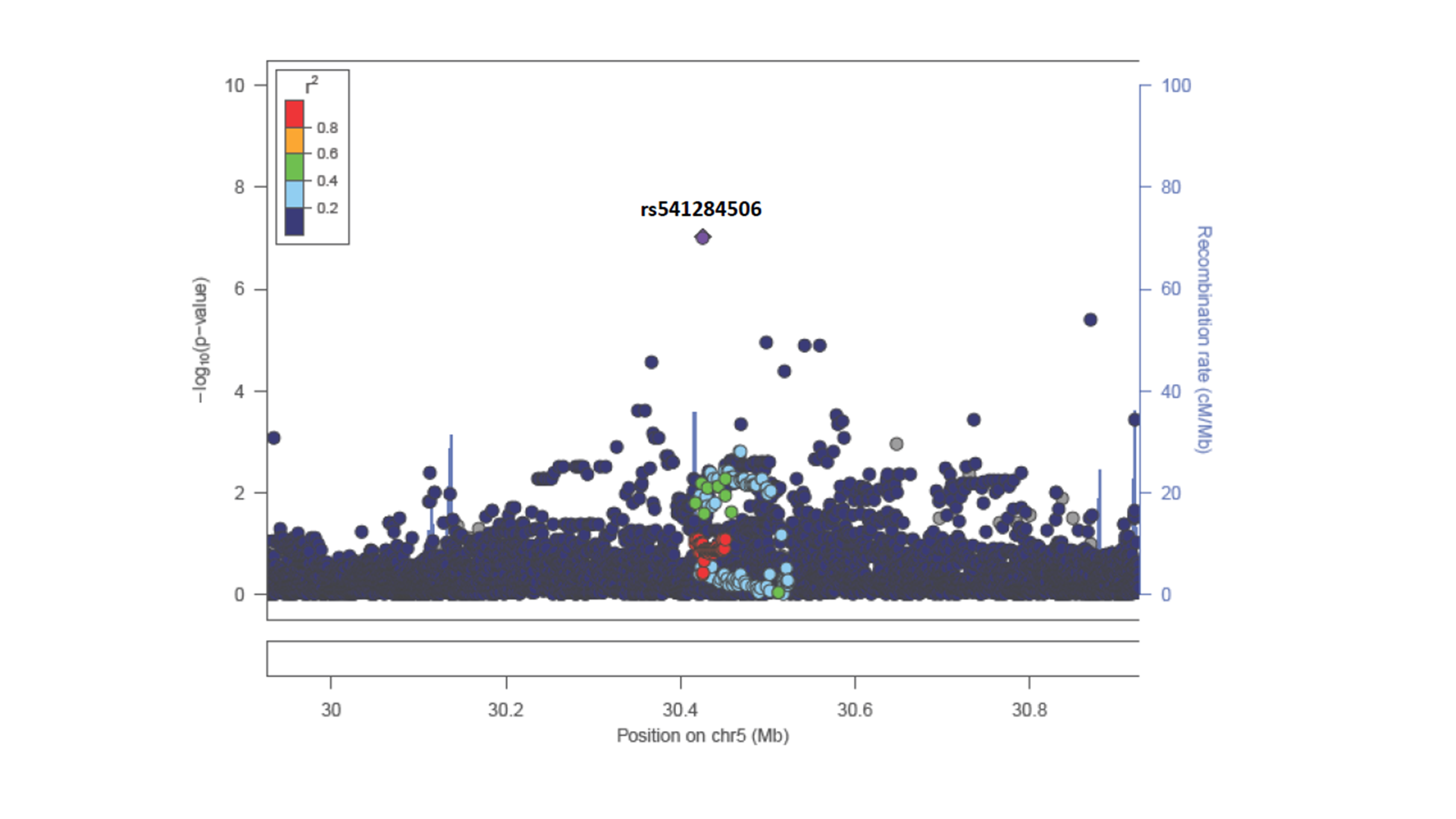

Supplement: Supplementary file 2 — Figure S1. Close up (1 Mb) Manhattan plot of Chr. 5 association peak (TIF 14502 kb) [file 40246_2019_206_MOESM2_ESM.tif]

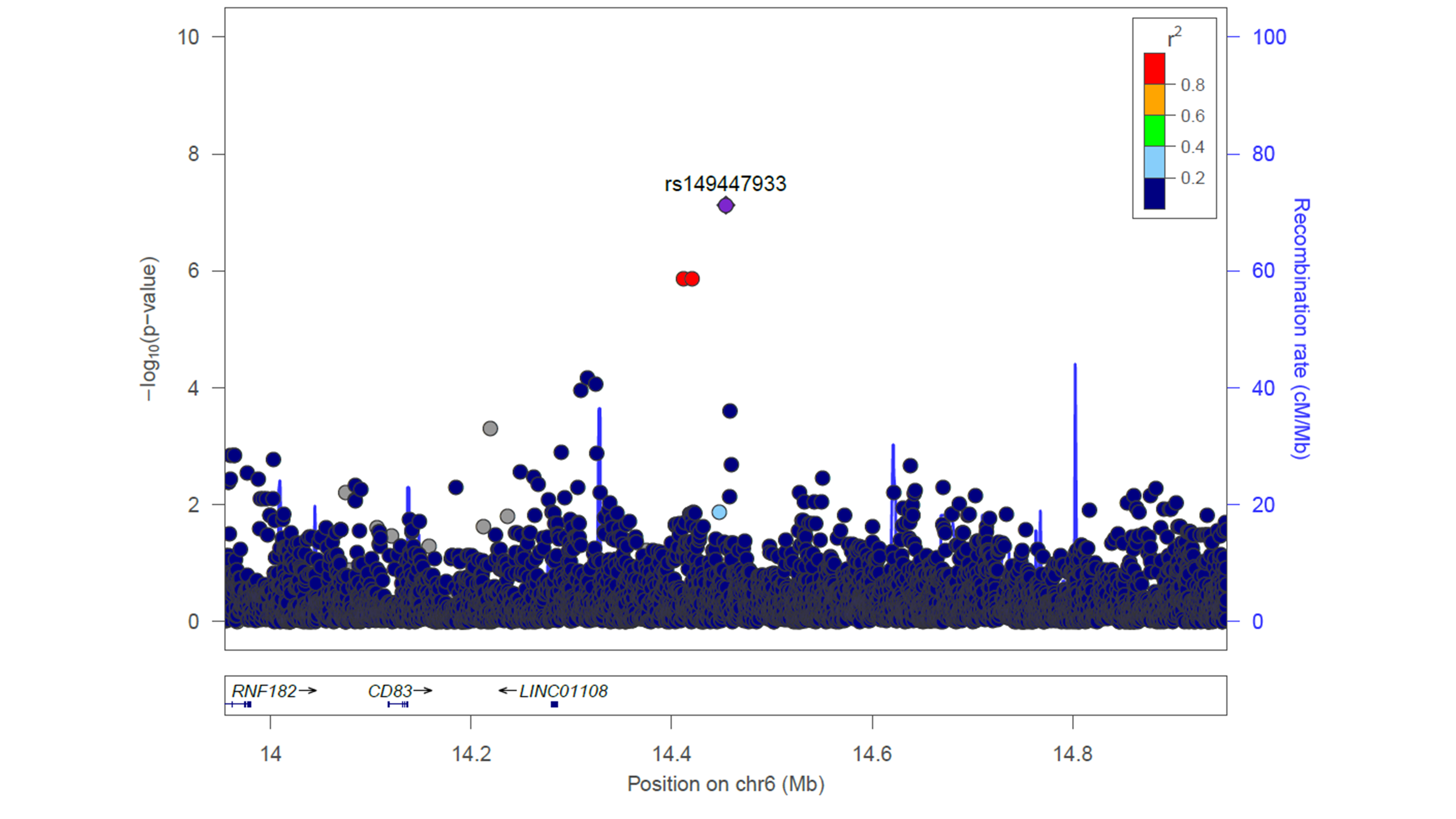

Supplement: Supplementary file 3 — Figure S2. Close up (1 Mb) Manhattan plot of Chr. 6 association peak (TIF 2869 kb) [file 40246_2019_206_MOESM3_ESM.tif]

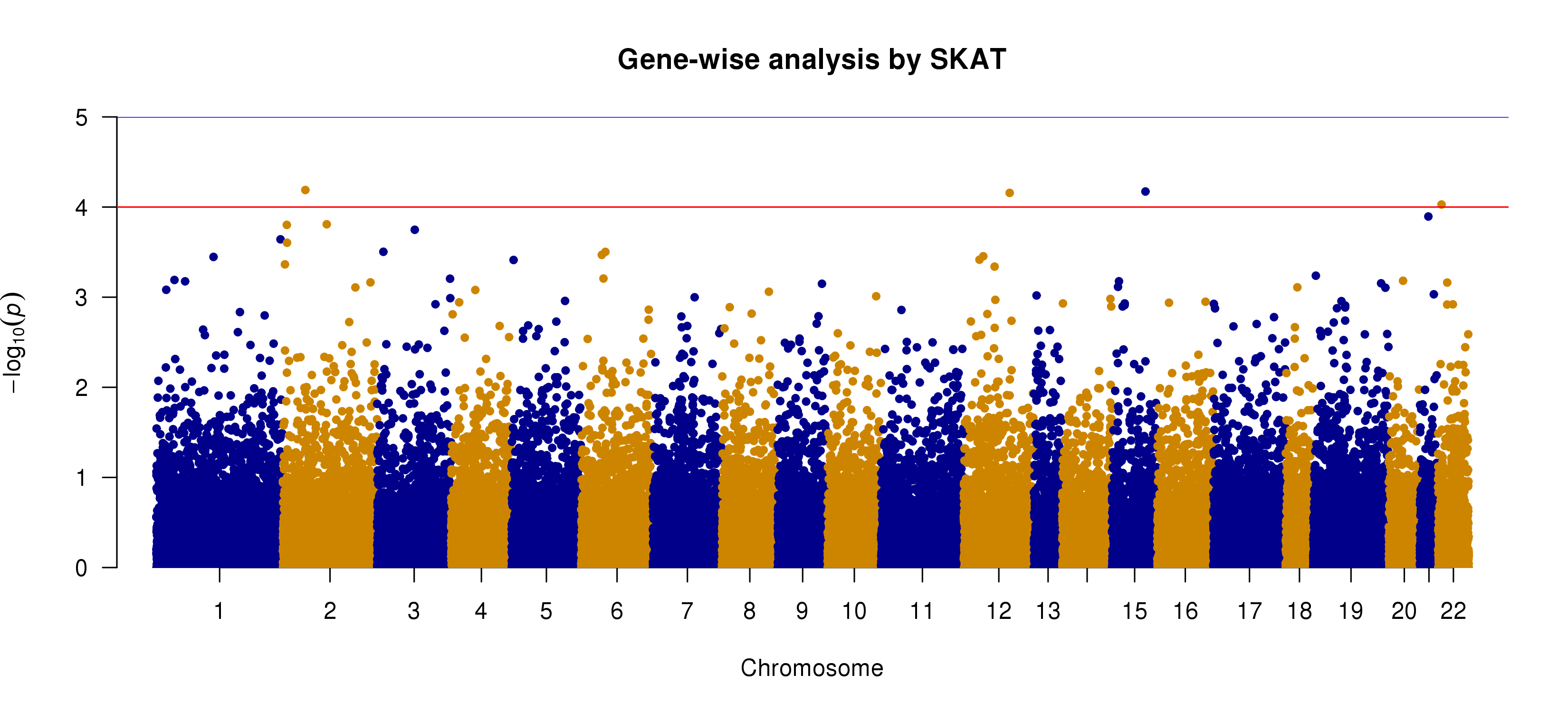

Supplement: Supplementary file 4 — Figure S3. Manhattan Plot of the gene-wise analysis of heart rate. Blue reference line: 1x10^-5^, Red reference line: 1x10^-4^. (TIFF 14100 kb) [file 40246_2019_206_MOESM4_ESM.tiff]

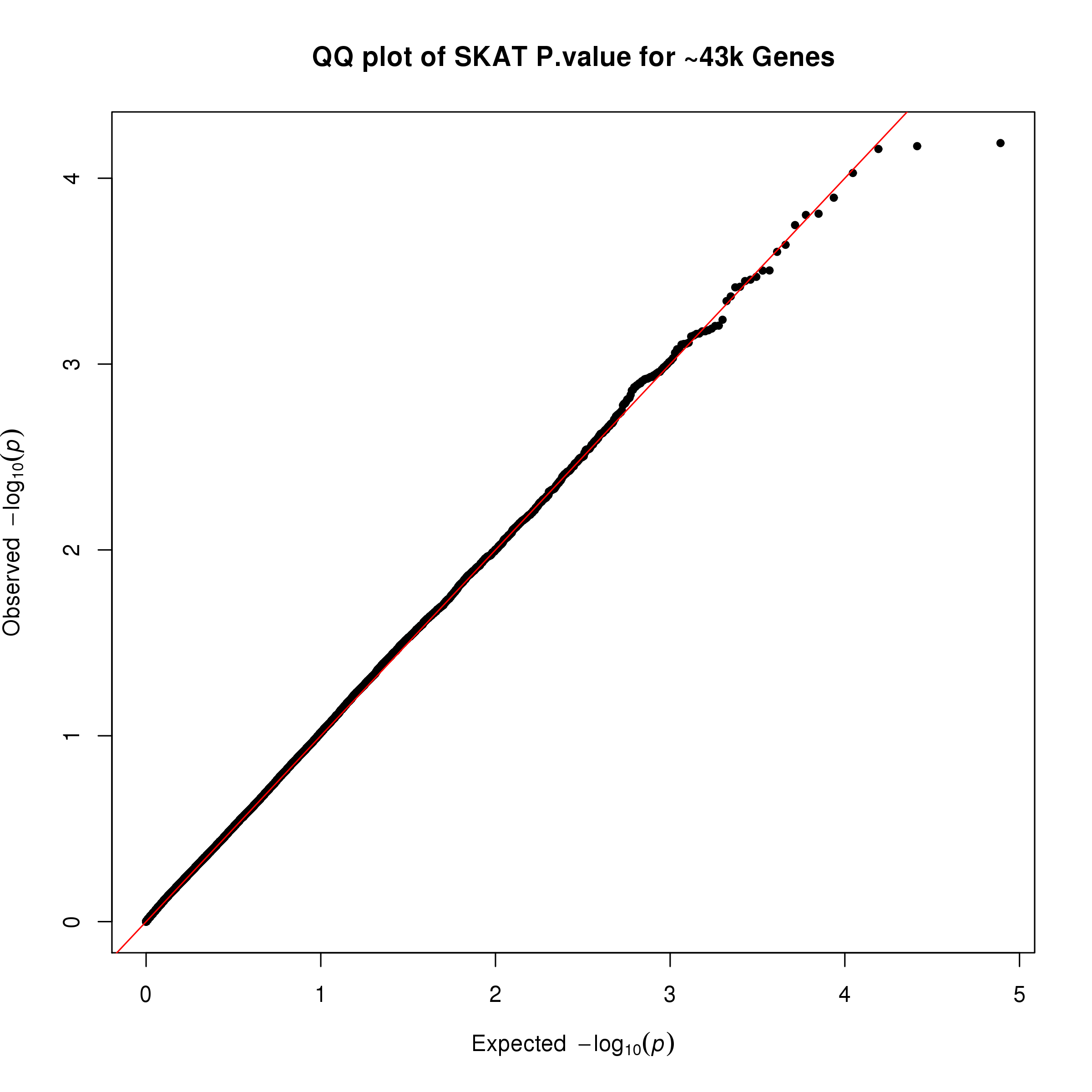

Supplement: Supplementary file 5 — Figure S4. Q-Q Plot of SKAT P values for approximately 43,000 genes from the gene-wise analysis of heart rate. (TIFF 16400 kb) [file 40246_2019_206_MOESM5_ESM.tiff]
